# Assessment of Sulfite Residues in Shrimp from Moroccan Fisheries: Implications for Food Safety and Regulatory Compliance

**DOI:** 10.3390/vetsci13060558

**Published:** 2026-06-05

**Authors:** Ghizlane Larif, Nourredine Bouchriti, Rachid Khatouf, Oleya El Hariri, Said Dahani

**Affiliations:** 1 Department of Veterinary Pathology and Public Health–Food Safety Unit, Hassan II Agronomic and Veterinary Institute, Rabat 10112, Morocco; n.bouchriti@iav.ac.ma (N.B.); khatoufrachid@gmail.com (R.K.); s.dahani@iav.ac.ma (S.D.); 2 Laboratory of Biochemistry, Biotechnology, Health and Environment, Department of Biology, Faculty of Science, University Ibn Tofail, Kenitra 14000, Morocco; oleyaelhariri@gmail.com

**Keywords:** sulfites, crustaceans, shrimp, melanosis, food safety, chemical residues, regulatory compliance, veterinary public health

## Abstract

Monitoring chemical residue levels in foods of animal origin is essential to ensure food safety and protect consumer health. Sulfites are commonly used in crustaceans to preserve quality; however, excessive levels may raise public health concerns, particularly for sensitive individuals. This study aimed to assess the compliance of shrimp from offshore and coastal fisheries in Morocco with current regulatory standards. A total of 60 samples were collected and analyzed for residual sulfite levels using the semi-quantitative strip test method. The results showed that sulfite concentrations ranged from 5 to 100 mg/kg in offshore samples, with a mean value of 41 mg/kg. Higher concentrations were observed in coastal samples, with an average of 172 mg/kg. Despite the observed differences between fishing origins, most samples complied with the established regulatory limits. These results support the importance of systematic monitoring programs within the fisheries supply chain to strengthen food safety control and contribute to veterinary public health protection.

## Simple Summary

The safety of seafood products is a major public health concern, as they can contain substances that may affect consumer health. Sulfites are chemical additives widely used to maintain the freshness and appearance of crustaceans such as shrimp. However, excessive sulfite levels can pose health risks, particularly for sensitive individuals. The aim of this study was to evaluate sulfite levels in shrimp from Morocco and to determine whether they comply with established safety standards. A total of 60 shrimp samples from offshore and coastal fisheries were analyzed to measure sulfite concentrations. The results showed that shrimp from offshore fishing contained sulfite levels ranging from 5 to 100 mg/kg, while higher concentrations were found in samples from coastal fisheries. Despite these differences, most samples remained within the recommended regulatory limits. These findings highlight the importance of ongoing monitoring of sulfite levels in crustaceans to protect consumer health and improve quality control practices across the fisheries supply chain.

## 1. Introduction

The crustacean sector plays an increasingly important role in national halieutic production, contributing significantly to the socio-economic development of the country. Supported by offshore and coastal fisheries, this activity ensures a steady supply of crustacean products to both local and international markets. However, due to their highly perishable nature, crustaceans are commonly treated with sulfites to prevent melanosis and maintain product quality during storage and commercialization.

To ensure food safety, the use of sulfites is regulated and monitored at the national level through self-monitoring systems implemented by professionals and official controls conducted by the veterinary services of the National Office for Food Safety (ONSSA). These control measures aim to limit the risks associated with excessive or inappropriate use of these additives within the food chain.

Sulfites are widely used food additives because of their antimicrobial and antioxidant properties; however, they are considered potential chemical hazards, particularly for sensitive individuals such as asthmatic consumers. Although long-term effects such as genotoxicity, carcinogenicity, or reprotoxicity have not been clearly established [1], toxicological studies have reported potential adverse effects, including neurophysiological responses in experimental models [2]. Despite their widespread use, limited data are available regarding sulfite levels and compliance in crustaceans marketed in Morocco.

Therefore, the objective of this study was to evaluate residual sulfite levels in shrimp from offshore and coastal fisheries in Morocco and to assess their compliance with current regulatory standards in relation to preservation practices applied on board vessels.

## 2. Materials and Methods

### 2.1. Sample Collection

This study was conducted to evaluate the occurrence of sulfites in crustaceans collected in Morocco. A total of 60 shrimp samples originating from offshore and coastal fisheries were analyzed. Among these samples, 30 were obtained from coastal fisheries and corresponded to pink shrimp (*Parapenaeus longirostris*), while the remaining 30 samples originated from offshore fishing operations and consisted of red shrimp (*Aristeus antennatus*).

All samples (200 g each) were collected at the landing stage during official veterinary sanitary inspection at two fishing ports in Morocco. Each port was associated with a specific fishing system: one was dedicated to coastal fisheries and the other to offshore (deep-sea) fisheries. Thirty samples were randomly collected from each port immediately after unloading and prior to commercialization, ensuring clear separation between the two production systems.

Samples were transported under refrigerated conditions and stored at −18 °C until analysis. Prior to testing, a preliminary sensory examination was systematically performed to assess organoleptic quality. The exact time between shrimp capture and sampling could not be precisely determined due to variability in fishing operations and onboard storage conditions.

### 2.2. Sensory Examination

A sensory examination of the frozen samples was performed before analysis. After thawing, the sensory quality of the samples was evaluated according to the procedures described in the Codex Standard for Frozen Shrimp (Codex Stan 92-1981) [3] and the Guidelines for the Sensory Evaluation of Fish and Shellfish in Laboratories (CAC/GL 31-1999) [4]. The following parameters were assessed:

- Dehydration and freezer burn (absent/present; superficial/deep);
- Odor (pleasant, sweet, rancid, etc.);
- Color (uniform pink, yellowish, greenish, brownish, etc.);
- Presence of foreign matter (absent/present).

### 2.3. Determination of Sulfur Dioxide (SO_2_) Residues

Residual sulfite levels were determined using a semi-quantitative test strip method based on a colorimetric reaction. Prior to analysis, shrimp samples were peeled, and the edible muscle was homogenized to ensure sample uniformity. The sulfite analysis was carried out in accordance with the manufacturer’s instructions (QUANTOFIX Sulfite 0–1000 mg/L, Macherey-Nagel, Düren, Germany), using the homogenized sample extract.

Each 200 g shrimp sample was thoroughly homogenized to obtain a uniform matrix. A natural aqueous extract was released due to the high moisture content of shrimp tissue and the hydrophilic nature of sulfites. This extract, corresponding to the soluble fraction of the homogenate, was directly used for sulfite screening. No external solvent or dilution was applied to preserve the native distribution of sulfites and maintain analytical sensitivity. The extract was analyzed immediately after sample preparation.

In this analysis, we employed QUANTOFIX^®^ test strips, which represent a commercially pre-calibrated semi-quantitative method based on defined sulfite standards provided by the manufacturer; no additional in-house calibration or validation against reference analytical methods (e.g., titration or chromatographic techniques) was performed in this study.

The analytical procedure consisted of immersing the test strip in the sample extract for approximately one second. The reactive zone of the strip was then compared with the colorimetric scale provided by the manufacturer to estimate sulfite concentration. The interpretation of the color change was performed after a standardized reaction time of 20 s to ensure reliable and consistent results. This method provides a rapid estimation of sulfite concentrations and is suitable for screening purposes.

### 2.4. Data Analysis

Due to the semi-quantitative nature of the colorimetric test strips, results falling between two concentration levels were assigned to the corresponding interval range. Sulfite concentrations were expressed in milligrams per kilogram (mg/kg), equivalent to parts per million (ppm) in solid food matrices. Descriptive statistics (minimum, maximum, mean, and standard deviation) were calculated to summarize the data and compare shrimp samples from offshore and coastal fisheries. Given the resolution of the test kit, all reported values were rounded to match the discrete concentration scale of the method.

Analytical data were processed using Microsoft^®^ Excel (Version 16.89.1, Microsoft 365; Microsoft Corporation, Redmond, WA, USA). Results were also compared with established regulatory limits to assess compliance of shrimp samples with current Moroccan standards.

## 3. Results

### 3.1. Sensory Evaluation Results

In accordance with the procedures specified in the Codex Stan 92-1981 standard for frozen shrimp by Codex Alimentarius [3] and the Guidelines for the Organoleptic Evaluation of Fish and Crustaceans in the Laboratory (CAC/GL 31-1999) [4], we based our assessment of the sensory quality of the samples on the following criteria:

- No dehydration or freezer burn;
- Pleasant, sweet smell;
- The color of the shrimp examined is generally uniform, with some samples from deep-sea fishing showing signs of blackening at the cephalothorax junction;
- No foreign matter in the samples.

Based on these factors, the samples were found to be organoleptically acceptable after thawing and prior to the start of our analyses.

### 3.2. Determination of SO_2_ Residues

The analysis was conducted on a set of 60 samples, including 30 pink shrimp (*Parapenaeus longirostris*) from coastal fishing and 30 red shrimp (*Aristeus antennatus*) from offshore fishing. The distribution of the samples, as well as the average count per kilogram, is presented in Table 1.

The analysis was performed in two consecutive phases, each comprising a distinct set of 30 samples. The results of the sulfur dioxide analysis in shrimp samples, carried out using the semi-quantitative test strip method, are summarized in Table 2.

The analysis of shrimp samples revealed variability in residual sulfur dioxide (SO_2_) levels according to their origin (Table 2). Offshore shrimp exhibited a mean concentration of 41 mg/kg, with values ranging from 5 to 100 mg/kg. In contrast, coastal shrimp showed higher concentrations, with a mean value of 172 mg/kg and a wider range of 50 to 500 mg/kg.

A higher standard deviation was observed in coastal samples (126 mg/kg) compared to offshore samples (28 mg/kg), indicating greater dispersion of sulfite levels within this group.

All analyzed samples fell within the working range of the method, and no exceedance of the upper detection limit or extrapolation beyond the calibrated scale was performed.

### 3.3. Class Distribution

Table 3 presents the class distribution of sulfite levels in shrimp samples from each fishing origin.

In offshore shrimp samples, residues were mainly distributed in the lower concentration classes, with the highest proportion observed in the 25–50 mg/kg range (36.7%), followed by the 10–25 mg/kg (26.7%) and 0–10 mg/kg (20.0%) classes. A smaller proportion of samples (16.7%) fell within the 50–100 mg/kg range, while no samples exceeded 100 mg/kg.

In contrast, coastal shrimp samples exhibited a shift toward higher residue levels, with the highest proportion of samples found in the 100–250 mg/kg class (40.0%), followed by the 25–50 mg/kg (26.7%) and 50–100 mg/kg (26.7%) classes. Only a small proportion of samples (6.7%) fell within the 250–500 mg/kg range, while no samples were observed in the 0–25 mg/kg range.

Overall, the distribution patterns indicate clear differences between fishing origins, with offshore samples predominantly characterized by low residue levels and coastal samples showing higher and more widely distributed sulfite concentrations. Regarding regulatory compliance, all offshore shrimp samples were below the maximum residue limit (300 mg/kg). In coastal samples, the majority of samples remained below this threshold, although two samples fell within the highest class (250–500 mg/kg), which may exceed the regulatory limit.

### 3.4. Relationship Between Blackening and Sulfur Dioxide Concentration

The sensory examination of shrimp samples revealed variability in coloration, ranging from bright pink to the presence of melanosis spots at the cephalothorax junction. This observation prompted the investigation of a potential relationship between residual SO_2_ levels and the degree of blackening. The results are summarized in Table 4.

**Figure 1:**
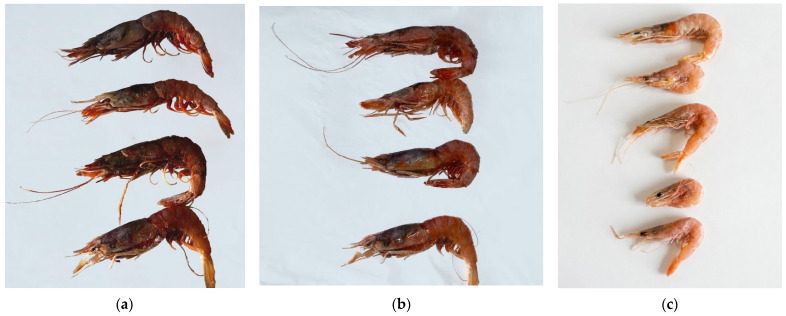
Shrimp samples classified according to sulfite residue levels determined by the strip test method: (**a**) 0–50 mg/kg; (**b**) 50–250 mg/kg; (**c**) 250–500 mg/kg.

The sensory examination of shrimp samples revealed general organoleptic compliance. However, some samples displayed dark spots localized at the cephalothorax junction, ranging from moderate intensity to pronounced melanosis. A relationship was observed between melanosis intensity and residual SO_2_ levels. Samples with pronounced melanosis were mainly associated with the 0–50 mg/kg class (Figure 1a), whereas samples with moderate signs of melanosis corresponded to the 50–250 mg/kg class (Figure 1b). In contrast, samples with SO_2_ levels in the 250–500 mg/kg range showed no visible signs of melanosis and exhibited a uniform pink coloration (Figure 1c).

## 4. Discussion

The present study provides a representative assessment of residual sulfur dioxide (SO_2_) levels in shrimp from different fishing origins, highlighting significant differences in sulfiting practices and their implications for organoleptic quality and consumer safety.

### 4.1. Variability of SO_2_ Levels According to Fishing Origin

The results clearly demonstrate that shrimp from coastal fishing exhibited substantially higher and more variable SO_2_ levels compared to offshore shrimp. This variability, reflected by the high standard deviation observed in coastal samples, suggests a lack of standardization in sulfite application practices. In contrast, the relatively low and homogeneous levels detected in offshore shrimp indicate better control of sulfiting procedures.

These findings are consistent with previous studies conducted in Morocco. Mean SO_2_ concentrations exceeding 250 mg/kg have been reported in pink shrimp from coastal sources [5], which is higher than the mean value observed in the present study but confirms the same trend of elevated sulfite levels in this category. Similarly, earlier investigations have shown that sulfite levels in crustaceans generally remained within regulatory limits, particularly in export-oriented products [6], which aligns with the lower concentrations observed in offshore shrimp in this study.

The observed differences may be explained by disparities in professional training, onboard handling practices, and regulatory oversight. Offshore fishing operations, often oriented toward export markets, are typically subject to stricter controls and better adherence to good manufacturing practices. In contrast, coastal fisheries may rely on more empirical and less controlled methods, contributing to the observed heterogeneity.

### 4.2. Compliance with Regulatory Standards and Food Safety Implications

The sulfite level in products intended for export to the European Union is regulated by Directive 95/2/EC of 20 February 1995 on food additives other than colors and sweeteners [7]. In contrast, products placed on the national market must comply with the provisions of Ministerial Order No. 1795-14 of 14 May 2014, which establishes the list and maximum limits of authorized food additives in foodstuffs, as well as mandatory labeling requirements [8]. According to this regulation, the maximum authorized limit for sulfites in crustaceans is set at 300 mg/kg. This regulatory framework ensures harmonization of food safety standards and transparency for consumers, particularly regarding the presence of sulfites in crustacean products.

Most of the analyzed samples complied with current regulatory limits for sulfite residues, as the majority of values remained below the national maximum limit of 300 mg/kg. However, the presence of samples within the 250–500 mg/kg range in coastal shrimp raises concerns regarding potential exceedances of the maximum authorized limits.

These findings are in line with previous reports highlighting occasional non-compliance in shrimp marketed locally. For example, extremely high SO_2_ levels (up to 2200 mg/kg) have been reported in shrimp from retail markets, suggesting that additional sulfite treatments may occur post-landing, particularly at the distribution or retail level [9].

Such practices may be associated with attempts to preserve the visual quality of shrimp by inhibiting melanosis, thereby extending shelf life and maintaining market value. However, excessive sulfite use poses potential health risks, especially for sensitive individuals, and may constitute a form of food fraud when used to mask product deterioration. In practice, the actual health risk should be considered in light of real consumption conditions. These results underline the importance of strengthening monitoring systems, particularly in downstream segments of the supply chain, including markets and retail points. Enhanced traceability and stricter enforcement of regulations are essential to ensure consumer protection.

These findings should be interpreted considering actual dietary exposure to sulfites. An exposure assessment based on commonly consumed foods in Morocco, including shrimp prepared according to traditional culinary practices, reported an average sulfite concentration of 42.2 mg/kg in shrimp, reflecting typical consumer exposure levels [10]. This value is comparable to the mean concentration observed in offshore shrimp in the present study, but remains markedly lower than the levels detected in some raw coastal samples. This discrepancy can be attributed to the impact of domestic processing, particularly washing and cooking, which are known to significantly reduce sulfite residues. This reduction is primarily related to the physicochemical properties of sulfites, which are highly water-soluble and relatively unstable under thermal and oxidative conditions. Indeed, cooking treatments have been shown to reduce sulfite levels by more than 50%, depending on the method applied [11]. Similarly, boiling promotes the leaching of sulfites into the cooking water, further contributing to their decrease [12]. These findings are consistent with previous studies indicating that sulfites are readily soluble in aqueous media and susceptible to degradation through heat and oxidation reactions [13,14]. Moreover, food processing conditions, including temperature and interactions with the food matrix, play a key role in determining sulfite stability and residual levels in food products [1]. Nevertheless, despite this reduction, the presence of high initial sulfite concentrations in some coastal samples suggests that residual levels after processing may still contribute significantly to overall dietary exposure, particularly in cases of excessive or non-compliant use.

The occurrence of sulfite-related non-compliance is not limited to the local context but reflects a broader international concern. Data from the European Rapid Alert System for Food and Feed (RASFF) database indicate that sulfites represented 4.2% of notifications in shrimp and prawn products between 2004–2008 and 2016–2017, with reports mainly issued by Italy for products originating from several countries, including Tunisia [15]. In addition, sulfite residues have been reported in imported shrimp marketed in the United States, where 43% of samples contained detectable levels between 10 and 100 mg/kg, although most remained within regulatory limits. Notably, none of the analyzed products declared sulfite treatment on their labels, highlighting deficiencies in labeling compliance and traceability within international supply chains [16].

Collectively, our findings highlight the importance of improving good handling and application practices of sulfites within the seafood supply chain, particularly at the level of fishing operations. The observed variability in residue levels, especially in coastal shrimp samples, suggests differences in sulfite application practices rather than systematic non-compliance. Therefore, targeted training of fishers on appropriate sulfite use and dose control may represent a more effective strategy to reduce variability and ensure consistent product quality. Strengthening traceability along the supply chain may further support the implementation of good practices and improve overall control of sulfite usage.

### 4.3. Interpretation of the Relationship Between Blackening and Sulfur Dioxide Levels

A clear inverse relationship was observed between SO_2_ concentration and the degree of melanosis. Samples with low SO_2_ levels (0–50 mg/kg) exhibited pronounced blackening, whereas higher concentrations (250–500 mg/kg) effectively inhibited melanosis, resulting in a uniform pink coloration. This observation is consistent with the well-documented role of sulfites as anti-melanosis agents. Sulfur dioxide acts by inhibiting polyphenol oxidase activity, thereby preventing the enzymatic browning process responsible for melanosis in crustaceans. However, these results also highlight a critical trade-off between visual quality and chemical safety.

While higher sulfite concentrations improve the aesthetic appeal of shrimp, they increase the risk of exceeding regulatory limits and potential adverse health effects. Conversely, lower concentrations may be insufficient to prevent melanosis, negatively affecting consumer acceptance.

These findings support the need for optimized sulfiting strategies that balance efficacy and safety. The importance of developing alternative anti-melanosis treatments that are both effective and safe, such as natural extracts or innovative preservation technologies, has been emphasized [17].

### 4.4. Implications for Professional Practices and Risk Management

The results suggest that sulfiting practices in coastal fisheries are not fully optimized. The predominance of intermediate SO_2_ levels (50–250 mg/kg), combined with high variability, indicates inconsistent application practices, likely associated with traditional sprinkling methods. Such approaches can result in heterogeneous distribution of sulfites, leading to both under- and over-treatment of products.

In contrast, more controlled application techniques, such as immersion or standardized spraying, may enhance dosing uniformity and regulatory compliance. This is consistent with findings reported for sulfite application in pink shrimp (*Parapenaeus longirostris*), where different application methods were compared under controlled conditions [18]. The authors showed that dipping ensured better compliance with residual SO_2_ limits, particularly at lower concentrations and shorter exposure times. Spraying provided intermediate performance, achieving a balance between melanosis inhibition and acceptable residue levels depending on formulation characteristics. Although dusting was the most effective method for delaying melanosis and improving sensory quality, it resulted in higher and more variable residual sulfite levels due to uneven distribution and prolonged product contact.

Beyond technological and regulatory considerations, sulfite exposure should also be addressed from a public health perspective. Epidemiological data from routine patch testing studies conducted across Europe and North America report sodium metabisulfite sensitization rates of approximately 1–3%, with values reaching up to 7% in some populations. Clinical relevance is frequently confirmed, ranging from 24% to over 60% in recent studies, with manifestations including contact dermatitis, urticaria, and, in severe cases, bronchospasm [19]. These findings suggest that although sulfite residues in food products are generally regulated and mostly remain within permissible limits, they may still represent a potential concern for susceptible individuals under conditions of repeated exposure or high dietary intake.

These observations support the implementation of a risk-based food safety management approach, allowing targeted surveillance of high-risk sectors such as coastal fisheries and informal distribution channels. Such an approach would enable more efficient allocation of control resources and improved mitigation of non-compliance risks [20].

Effective management of sulfite-related hazards also requires a co-regulatory framework involving both industry stakeholders and competent authorities [21]. Operators bear primary responsibility for ensuring compliance with maximum permitted concentrations throughout the production chain, from capture to commercialization. This necessitates the implementation of documented self-monitoring systems, including analytical controls and corrective actions when deviations occur. In parallel, the National Office for Food Safety (ONSSA) plays a central role in official surveillance through inspections, sampling, and laboratory analyses conducted in accredited facilities to verify regulatory compliance.

Finally, capacity building and training of operators represent essential components of risk mitigation. Improving knowledge of sulfite use, regulatory thresholds, and good handling practices can significantly reduce misuse. In this context, the involvement of professional associations may facilitate knowledge transfer and strengthen communication between stakeholders, thereby enhancing overall compliance and food safety governance.

### 4.5. Limitations and Perspectives

The use of semi-quantitative test strips in this study provided a rapid and practical screening tool for sulfite detection in shrimp samples under controlled post-collection laboratory conditions. Such approaches are widely used in routine monitoring and have also been adapted for consumer-level applications, highlighting their versatility across different use contexts [22].

Recent analytical developments have significantly expanded the available toolbox for sulfite detection. Among them, surface-enhanced Raman spectroscopy (SERS) enables non-destructive identification of sodium metabisulfite residues on shrimp surfaces, with detection limits as low as 0.31 mg/kg, making it suitable for in situ screening applications [23]. In addition, enzymatic biosensor systems such as the BIOFISH 300 SUL, recently validated as an AOAC Official Method of Analysis, allow rapid and reliable quantification of total sulfites in less than 3 min, even at concentrations close to regulatory thresholds [24].

Although these emerging technologies offer strong potential for operational monitoring and rapid decision-making, they remain complementary to confirmatory reference methods such as the optimized Monier–Williams procedure, which is still required for regulatory enforcement. Consequently, an integrated analytical strategy combining rapid screening tools with reference confirmatory methods is recommended to ensure both efficiency and analytical robustness across the seafood supply chain.

Future research should focus on investigating the influence of handling practices, storage conditions, and processing methods on sulfite variability in shrimp. In addition, exploring alternative anti-melanosis strategies, including safer and more sustainable compounds, would represent a promising avenue to reduce reliance on sulfites while maintaining product quality.

## 5. Conclusions

The present study provides quantitative evidence of residual sulfite levels in shrimp from Moroccan fisheries, highlighting clear differences according to the origin of the samples. Offshore shrimp exhibited relatively low sulfite concentrations (mean: 41 mg/kg; range: 5–100 mg/kg), whereas coastal shrimp showed substantially higher levels (mean: 172 mg/kg; range: 50–500 mg/kg), indicating greater variability in sulfiting practices within coastal supply chains.

Overall compliance rates reached 100% for offshore samples and 93.33% for coastal samples, suggesting that sulfite application onboard fishing vessels is generally well controlled. Nevertheless, the higher concentrations and broader variability observed in coastal shrimp emphasize the need for improved standardization of handling and preservation practices throughout the distribution chain.

Although sulfites remain widely used due to their technological effectiveness in preventing enzymatic melanosis and preserving product freshness, their potential to induce adverse reactions in sensitive individuals and the possibility of cumulative dietary exposure continue to raise food safety concerns. These findings support the importance of maintaining strict monitoring programs and mandatory sulfite labeling to ensure consumer protection.

From a risk management perspective, strengthening good handling practices, operator training, and monitoring systems throughout the seafood supply chain remains essential to minimize excessive sulfite exposure and ensure both product quality and public health safety.

## Figures and Tables

**Table 1:** Sample distribution by origin, species, and count per kilogram.

Origin	Species	Number of Samples	Count Per kg ^1^
Offshore fishing	Red shrimp	30	80
Coastal fishing	Pink shrimp	30	130

^1^ Number of pieces per kilogram (pieces/kg).

**Table 2:** Residual SO_2_ levels in the analyzed crustaceans.

Origin of Samples	Number	Mean	Standard Deviation	Range (Min–Max)
Offshore fishing	30	41	28	5–100
Coastal fishing	30	172	126	50–500

**Table 3:** Class distribution of sulfite residue levels in shrimp samples according to fishing origin.

	Origin of Samples			
Sulfite Residue Classes ^1^	Offshore Fishing		Coastal Fishing	
	Number	Percentage (%)	Number	Percentage (%)
0–10 mg/kg	6	20.0	0	0.0
10–25 mg/kg	8	26.7	0	0.0
25–50 mg/kg	11	36.7	8	26.7
50–100 mg/kg	5	16.7	8	26.7
100–250 mg/kg	0	0.0	12	40.0
250–500 mg/kg	0	0.0	2	6.7

^1^ Sulfite residue classes correspond to the concentration ranges of the QUANTOFIX^®^ semi-quantitative scale. Class intervals include the upper limit (e.g., 0–10 mg/kg includes values ≤ 10 mg/kg; 10–25 mg/kg includes values > 10–≤25 mg/kg, etc.). The 500–1000 mg/kg range was not observed in this study.

**Table 4:** Relationship between residual SO_2_ levels and melanosis degree in shrimp.

SO_2_ Level	Blackening Degree
0–50 mg/kg	++
50–250 mg/kg	+
250–500 mg/kg	-

++, pronounced melanosis; + moderate melanosis; -, absence of visible melanosis.

## Data Availability

The original contributions presented in this study are included in the article. Further inquiries can be directed to the corresponding author.

## Abbreviations

The following abbreviations are used in this manuscript:

EFSA	European Food Safety Authority
EU	European Union
ONSSA	National Office for Food Safety
ppm	Parts per million
RASFF	Rapid Alert System for Food and Feed
SO_2_	Sulfur dioxide
